# Integrating Social Capital Theory, Social Cognitive Theory, and the Technology Acceptance Model to Explore a Behavioral Model of Telehealth Systems 

**DOI:** 10.3390/ijerph110504905

**Published:** 2014-05-07

**Authors:** Chung-Hung Tsai

**Affiliations:** Department of Health Administration, Tzu Chi College of Technology, 880, Section 2, Chien-Kuo Road, Hualien 970, Taiwan; E-Mail: tsairob@tccn.edu.tw; Tel.: +886-3857-2158; Fax: +886-3857-8941

**Keywords:** telehealth, social trust, institutional trust, social participation, technology acceptance model, system self-efficacy

## Abstract

Telehealth has become an increasingly applied solution to delivering health care to rural and underserved areas by remote health care professionals. This study integrated social capital theory, social cognitive theory, and the technology acceptance model (TAM) to develop a comprehensive behavioral model for analyzing the relationships among social capital factors (social capital theory), technological factors (TAM), and system self-efficacy (social cognitive theory) in telehealth. The proposed framework was validated with 365 respondents from Nantou County, located in Central Taiwan. Structural equation modeling (SEM) was used to assess the causal relationships that were hypothesized in the proposed model. The finding indicates that elderly residents generally reported positive perceptions toward the telehealth system. Generally, the findings show that social capital factors (social trust, institutional trust, and social participation) significantly positively affect the technological factors (perceived ease of use and perceived usefulness respectively), which influenced usage intention. This study also confirmed that system self-efficacy was the salient antecedent of perceived ease of use. In addition, regarding the samples, the proposed model fitted considerably well. The proposed integrative psychosocial-technological model may serve as a theoretical basis for future research and can also offer empirical foresight to practitioners and researchers in the health departments of governments, hospitals, and rural communities.

## 1. Introduction

Demographic structures change as society changes. Developed countries worldwide are experiencing aging problems. Statistics by the Ministry of Interior, Executive Yuan of the R.O.C. (Republic of China, Taiwan) revealed that the senior citizen population was 7% of the total population in Taiwan in 1993. Taiwan has become an aging society, as defined by the World Health Organization. This percentage reached 10.9% at the end of 2011, and the aging index increased from 42.3% to 72.2%. The population aged 65 or older is estimated to exceed 20% by 2026. Taiwan would then become a super-aged society. In addition, the senior citizen population is estimated to be 36.97% of the total population by 2051; in other words, one senior citizen aged 65 or older for every three citizens.

With the phenomenon of the aging of the population, some crucial issues with seeking medical services have been emerged, including emergency medical care, life care when physical and mental functions are lost, chronic diseases, and so on. These care issues have revealed the increasing demands of long-term care and senior health management. Telhealth is considered one possible solution to answer these demands by providing cost-effective care through the use of information technology and biomedical technology.

Under the promotion of the National Information Infrastructure (NII), Executive Yuan, R.O.C. (Taiwan), Taiwan’s government has accumulated experience in implementing Telemedicine/Telecare/Telehealth Policy for more than 10 years with focus in supporting people living in offshore islands and mountain areas. Since 1995 the Department of Health, Executive Yuan, R.O.C. (Taiwan) has been promoting Telemedicine Pilot Program to remedy the problem of inadequate medical resources in rural area. The applications of Telemedicine can be categorized into three areas—Teleconsultation, Telediagnosis and Teleeducation. Since 2007 the Department of Health has commissioned Information and Communications Laboratories, Industrial Technology Research Institute to develop Telecare Pilot Program. It is aimed at developing Community-based, Home-based, Institution-based Telecare service models and their application systems with user-friendly human/machine interface through introduction and application of the information and communication technology, thus further establishing Telecare Information Integration Platform. In 2008 Telecare Quality and Service Improvement Program was launched. An integrated and continual network of Telemedicine was developed based upon two original telemedicine models consisting of Home/Community-based and Institution-based models. Meanwhile Ministry of Economic Affairs began to launch two famous telehealth projects. The M-care project, which was one of Mobile Taiwan Applications promotion projects (M-Taiwan), was one of the initial telehealth projects in 2006. The purpose of this project was to deliver health care into remote country to improve residents’ health. In 2007 the U-Care project was launched. The scope of this project was to integrate various industries to create a valuable and innovative business model of telehealth care service. Owing to the advantages of telecommunication technology and medical care, the Taiwanese government in 2008 included telecare and telehealth as a development project in the emerging service industry [1]. 

The R.O.C. government is promoting relevant health care plans to resolve the weakening care-giving capability of families from the increase of nuclear family, which is made up of the parents and their children, and to manage increasing demands for long-term health care services in an aging society. Information technology (IT) has gradually been integrated into health care services. For residents in rural areas, resources in the community may be effectively combined with medical care and localized through community-based telecare services, helping people self-monitor their physiological conditions and develop healthy behaviors.

Telemedicine has been defined as the electronically transmitted rapid exchange of medical information between sites of clinical practice for the purposes of relief and/or education. Telemedicine includes diagnosis, treatment, monitoring, and education of patients by using systems that allow ready access to expert advice and patient information no matter where the patient or relevant information is located [2]. That is, telemedicine emphasize the use of information and communication technologies (ICT) to provide clinical services to patients in remote areas, for instance, video conferencing with specialists, remote medical diagnoses, and the digital transmission of medical imaging data. Telecare refers to technology that allows patients to stay safe and independent in their own homes. It is characterized by remote, automatic, and passive monitoring of personal health and safety, and home environment [3]. These devices include fall detection systems, lifestyle monitoring devices and home videophones. Telehealth refers to the remote exchange of data between a patient (usually at home) and healthcare professionals (at a monitoring center) to assist in the management of an existing long-term condition [3]. The service scope of telehealth is more broadly than telemedicine and telecare. The service scope of telehealth is more broadly than telemedicine and telecare. It also includes administration and training in addition to clinical services [4]. Some telehealth programs’ goals now have extended beyond chronic care to include acute care management, health promotion, and disease prevention [5]. Telehealth allows for such things as remote doctor-patient consultations (telemedicine), remote monitoring of vital signs (e.g., blood pressure), and health education services. The use of telehealth that can facilitate the assessment of symptoms reported by patients themselves, may enhance effective symptom management in medical care and provide a means to overcome identified barriers to home care, improving the patients’ experience of care [6]. The purpose of target systems of this currently study is not only to provide the rural residents with remote clinical services, but also self-management of health and disease prevention. To avoid confusion, this study will use the term telehealth throughout the whole article. 

The Technology Acceptance Model (TAM) has been found more favorable in many studies [7]. Many studies of TAM have confirmed its robustness and reliability to predict and explain IS acceptance behavior [8]. Some studies have also utilized TAM to investigate patients’ adoption of healthcare information systems and found that TAM provides an appropriate theoretical basis [1,9,10,11]. Despite the widespread use of TAM in practice, little attention is paid to extending TAM to better understand the adoption of telehealth systems. Davis [12] also proposed that additional factors should be added to the TAM based on the study context. Specially, the goal of telehealth is to extend medical services provided by remote hospitals and to provide early diagnosis and early treatment through patients’ self-management of health. Telehealth is not only an innovative information system, but also health care delivery systems. Therefore, there is a need for research to expand TAM to include important variables and examine the relationships among those variables and eventual telehealth acceptance behavior.

Social capital refers to the features of social structure, such as networks, norms, and social trust, which facilitate coordination and cooperation for mutual benefits [13]. Social capital has been proven to be critical for maintaining population health [14]. In addition, the self-efficacy concept of the social cognitive theory (SCT) of Bandura [15] has been used to understand the behavior and performance of people in various activities. From an empirical standpoint of computer/information technology context, self-efficacy has been found to be an important determinant of the perceptions of users about such technologies [16]. Rahimpour *et al.* [10] also suggested system self-efficacy should be critical important in the acceptance of telehealth. 

Accordingly, the objective of this study is to integrate the theoretical perspectives of the TAM, social capital theory, and social cognitive theory to theoretically develop and empirically examine the behavioral model of residents in a rural community in using telehealth and to understand the relationships among those crucial factors that affect their behavioral intention of telehealth. Furthermore, the structural equation modeling (SEM) was used to examine the psychometric properties of questionnaires and validate the proposed model and hypotheses. The insights of these findings can be used for the further implementation of telehealth systems and may contribute to the future academic studies in this field.

## 2. Literature Review

### 2.1. Telecare and Telehealth

Telecare is defined as “the continuous, automatic and remote monitoring of real time emergencies and lifestyle changes over time in order to manage the risks associated with independent living” [17]. Telecare encompasses a wide range of equipment (e.g., detectors, monitors, alarms, and pendants) and services (e.g., monitoring, call centers, and response). Telecare equipment is used to support people in their homes and is tailored to meet their specific needs. Telecare services range from a basic community alarm service that reacts to an emergency and transmits a response to an integrated system that includes detectors and monitors (e.g., falls, fire, and gas), triggering a warning to a response centre. Telecare involves using information and communication technologies to transfer medical information for diagnosing and treating patients in their homes [17].

In 2007, the Department of Health of Executive Yuan in Taiwan commissioned telecare projects that established three telecare models: community-, home-, and institution-based telecare [18]. This project integrated medical care, medical equipment, information communication technology, and security protection to provide a model of holistic, continued, accessible, and digital health care services. Telecare services include: (1) physiological information retrieval, such as body temperature, heart rate, respiratory rate, and blood pressure, blood sugar, and blood oxygen levels; (2) communication and collaboration of health care services, such as urgent calls, transmission of abnormal alarm signals, and notice to revisit; and (3) assistance of health self-management, such as collecting changes of physiological information daily, self-management and follow-up, and early prevention.

Proponents of telecare suggest that it can enable older people to live safely and independently. Three generations of telecare systems can be identified. The first generation of telecare systems was technically simple because the systems comprised no embedded intelligence and relied entirely on the user to activate calls. The second generation systems have all of the features of the first generation but also provide some level of intelligence and automatic detection under limited alert conditions. The third-generation systems provide additional support capabilities, such as lifestyle monitoring or reassurance and the introduction of virtual neighborhoods [19].

Telehealth refers to the remote exchange of data between a patient (usually at home) and healthcare professionals (at a monitoring center) to assist in the management of an existing long-term condition [3]. The service scope of telehealth is more broad than those of telemedicine and telecare. It also includes administration and training in addition to clinical services [4]. Some telehealth programs’ goals now have extended beyond chronic care to include acute care management, health promotion, and disease prevention [5]. Telehealth has been considered a partial solution to the problems of delivering health care to remote areas as well as to areas underserved by health care professionals [20]. The devices monitor vital signs of patients (or rural residents) include blood pressure, blood glucose, blood oxygen, and weight. Telehealth system is currently being trailed to improve older people’s ability to remain within their homes as long as possible, with access to care and support services that enable them to do this safely [11]. The improved access and quality of clinical care available to rural residents through telehealth may contribute to decreasing the urban-rural health disparities. Also, the professional development opportunities and support from specialists through the use of telehealth may contribute to improved rural medical workforce recruitment and retention [4]. Besides, the use of telehealth that can facilitate the assessment of symptoms reported by patients themselves may enhance effective symptom management in medical care and provide a means to overcome identified barriers to home care, improving the patients’ experience of care [6].

### 2.2. Technology Acceptance Model

The Technology Acceptance Model (TAM) is a frequently used behavioral model for predicting and explaining IT usage [21]. The TAM identifies two relevant beliefs, namely perceived ease of use and perceived usefulness. Perceived ease of use is defined as the extent to which a person believes that using a system is free of effort, whereas perceived usefulness is defined as the extent to which a person believes that using a system enhances job performance [22]. According to the TAM, using IT is influenced by behavioral intention to use the IT, and behavioral intention is determined jointly according to perceived ease of use and perceived usefulness. Perceived usefulness is also influenced by perceived ease of use and external variables. In the past decade, the TAM has been widely applied in practice, extended in academics, and empirically tested in the field of information management. Based on the TAM, some studies have investigated the perceptions of patients toward health care IS [23]. In addition, some studies have utilized TAM to investigate patients’ adoption of healthcare information systems and found TAM provides an appropriate theoretical basis [1,9,10,11].

In this study user adoption was examined by intention to use telehealth systems rather than actual adoption. There are a couple of reasons as follows. Firstly, owing to the difficulties in interpreting the multidimensional aspects of “use” (mandatory *versus* voluntary, informed *versus* uninformed, effective *versus* ineffective, and so on), DeLone and McLean [24] suggested “intention to use” may be a worthwhile alternative measure in some contexts. Secondly, Davis, Bagozzi, and Warshaw [22] found that behavioral intention to use the system has a positive significantly effect on usage. Owing to the strong causal link between behavioral intention and actual behavior, use of behavioral intention as the dependent variable is not a serious limitation [25]. A number of prior studies either suggested or indicated that behavioral intention to use the system is a reasonable indicator of future system usage [26]. Finally, actual use can be replaced by intention to use when the technology is still undergoing development, has a limited number of users, and when the objective of the research is to predict future use [27]. Telehealth is still at the early development stage, characterized by limited technology adoption and use [28]. Recently, many telehealth studies still used the intention to use variable as proxy of adoption [1,9,10].

### 2.3. Social Capital and the Technology Acceptance Model

Bourdieu [29] proposed that social capital is the aggregate of actual or potential resources, which are linked to possession of durable networks of relatively institutionalized relationships of mutual acquaintance or recognition. Coleman [30] stated that social capital includes several aspects of social structure and facilitates certain actions by individuals within the structure. Putnam [13] argued that social capital indicates the features of social structure, such as networks, norms, and social trust, which facilitate in coordinating and cooperating for mutual benefits. Woolcock [31] also stated that social capital includes the information, trust, and norms of reciprocity inherent in social networks. Nahapiet and Ghoshal [32] defined social capital as the sum of the actual and potential resources embedded within, available through, and derived from the network of relationships maintained by an individual or social unit. 

In sum, social capital focuses on the fitness of the individuals and their personal relationships. Social capital can be viewed as a facilitator of social structure for certain actions of individuals, which benefit both the individuals and the organizations [13,29,30]. It mainly deals with interactions between people [33]. Similar to all other forms of capital, social capital has common features: (1) it is a long-lived asset, (2) it is both “appropriable” and “convertible”, (3) it can either be a substitute for or complement other resources, (4) it needs maintenance, (5) some forms of social capital are “collective goods” [34]. 

Social capital may contextually affect individual health through several mechanisms, such as promoting rapid diffusion of health information, increasing the likelihood that healthy norms of behavior are adopted, exerting social control over deviant health-related behavior, increasing access to local services and amenities, and providing affective support and acting as the source of self-esteem and mutual respect [35]. In addition, social capital theory suggests that social capital satisfies a necessary condition for knowledge exchange. Lindstrom and Janzon [36] also proposed that social capital is a social and contextual factor. A society exhibiting high levels of social capital typically encounters a high level of civic engagement, social participation, social trust, institutional trust, and interpersonal reciprocity. Nummela *et al.* [37] indicated that the core aspects of social capital, such as trust and social participation, have health-protective effects. 

Social capital has mostly been measured as patient trust or social participation/social networks [13,37]. Patient trust refers to the expectation that an individual or institution acts competently, fairly, openly, and considerately. Mohseni and Lindstrom [38] argued that patient trust could be divided into vertical trust in the institutions of society (institutional trust) and horizontal trust or generalized trust in other people (social trust). The health care system is an institution. Institutional trust enables patients to trust providers without any personal knowledge of the health workers [39]. Patient trust in health care providers (*i.e.*, social trust) has been associated with the clinical or technical competence of the providers, interpersonal quality of care, and concern for the person rather that the disease only. In addition, patient trust in the health care system (*i.e.*, institutional trust) also encourages the use of services, submission to treatment, and patient compliance [38]. 

Social participation is an observable feature of social capital that can be measured according to the density of an organization in a geographical area or by the level of participation by respondents in formal or informal social activities within a society [13,38,40]. Social participation provides the channels through which people can be recruited to perform good deeds and social networks can be used to foster norms of reciprocity that encourage people to consider the welfare of others [13,41]. 

Several previous empirical studies have supported integrating social capital with the TAM. Reid [42] integrated trust with the TAM, arguing that trust relates to perceived ease of use and perceived usefulness. Gefen, Karahanna, and Straub [43] also proved that institutional trust positively affects perceived ease of use and perceived usefulness. Pavlou [44] proposed the e-commerce acceptance model, and the findings support the modified TAM with which trust was integrated. Chow and Chan [45] suggested that social participation provided increased opportunities for interpersonal contact. People reported positive feelings toward sharing ideas and resources with those whom they developed close relationships. Chow and Chan [45] also showed a positive association between social participation and attitudes toward knowledge sharing.

The utility of telehealth is that it could improve older people’s ability to remain within their homes as long as possible, with easier access to care and more support services that enable them to do this safely. Trust enable residents to believe that the healthcare providers or the health institution (hospital) have enough ability, benevolence, and integrity to provide expected positive outcome to them. Similarly, trust enable residents to believe that the healthcare providers or the health institution (hospital) will provide required assistances and supports (e.g., training courses, consultants, professional staffs, and user guide handbook) to improve perceived ease of use of telehealth. 

Also, if the community residents show higher levels of social participation, then they will become more actively involved in associational activities. These associations often offer opportunities for residents to acquire, maintain, or improve the skills of using telehealth, which in turn, may improve perceived ease of use of telehealth. Besides, higher levels of social participation enable residents to share more feeling, health knowledge and resources about telehealth, which in turn, may also promote perceived usefulness of telehealth.

### 2.4. System Self-efficacy and the Technology Acceptance Model

Social cognitive theory (SCT) is a theoretical framework for analyzing human motivation, thought, and action that embraces an interact model of causation in which behavior, cognition and other personal factors, and environmental influences all operate as interacting determinants that influence each other bidirectionally [15,46]. Self-efficacy is a key element in SCT and refers to the judgments of people regarding their capabilities to organize and execute courses of action required to perform a task. Self-efficacy affects the behaviors that people choose to perform, the amount of effort they are prepared to exert, and the length of their persistence to overcome obstacles [8,15,47]. According SCT, self-efficacy is a major determinant of an individual’s task performance and has been found to have diverse psychological and behavioral effects in many areas of human psychosocial functioning [15,46]. From an empirical standpoint of computer/information technology context, self-efficacy has been found to be an important determinant of the perceptions of users about such technologies [16]. 

Adapted from the general concept of self-efficacy, Compeau and Higgins [48] defined computer self-efficacy as “people’s judgments about their abilities to use a computer system successfully”. In the telehealth context, this innovative system should be a special application of computer systems. Rahimpour *et al.* [10] conceptualized the self-efficacy of a telehealth system as the judgment of a person’s capability to use the system. Although most of the participants in that study exhibited high levels of usage intention, they also expressed low levels of confidence in using the system. Therefore, Rahimpour *et al.* suggested that system self-efficacy is likely to be crucial and should be incorporated into the TAM to develop an extended model for the acceptance of telehealth by patients. 

Venkatesh and Davis [16] and Venkatesh [49] proposed that users will anchor their ease of use perceptions to the more abstract belief about their abilities to put computer technologies to use (*i.e.*, system self-efficacy), and will require direct interaction with a system in order to be able to form perceptions about more tangible aspects of the system. These two studies have showed that system self-efficacy is a strong determinant of perceived ease of use before hands-on experience. Many previous empirical studies have also found system self-efficacy had a positive effect on perceived ease of use [8,50]. 

In addition, some studies have indicated that significantly positive relationships exist between system self-efficacy and outcome expectation [51], which refers to the extent to which the behavior of people, once successfully executed, is believed to be linked to valued outcomes (*i.e.*, perceived usefulness) [22]. Some studies have found a positive relationship between system self-efficacy and perceived usefulness [8,49,50].

Accordingly, this study proposes the following hypotheses:

*H1: Social trust positively affects perceived ease of use in a telehealth context*.
*H2: Social trust positively affects perceived usefulness in a telehealth context*.
*H3: Institutional trust positively affects perceived ease of use in a telehealth context*.
*H4: Institutional trust positively affects perceived usefulness in a telehealth context*.
*H5: Social participation positively affects perceived ease of use in a telehealth context*.
*H6: Social participation positively affects perceived usefulness in a telehealth context*.
*H7: System self-efficacy positively affects perceived ease of use in a telehealth context*.
*H8: System self-efficacy positively affects perceived usefulness in a telehealth context*.
*H9: Perceived ease of use positively affects perceived usefulness in a telehealth context*.
*H10: Perceived ease of use positively affects usage intention in a telehealth context*.
*H11: Perceived usefulness positively affects usage intention in a telehealth context*.

**Figure 1:**
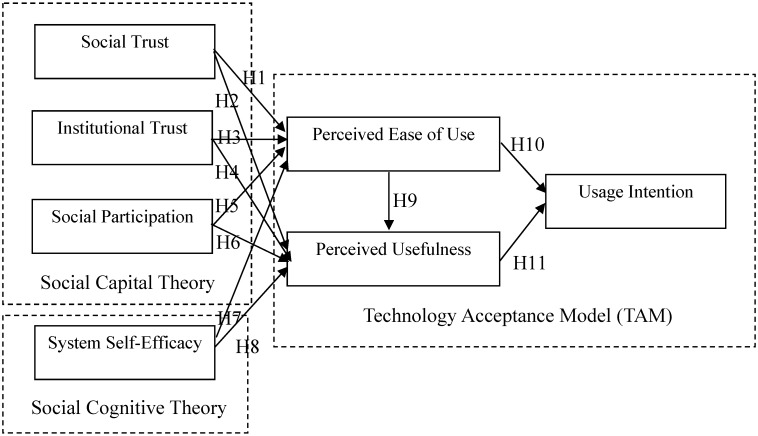
The Proposed Research Model.

The previous arguments support the proposed research model illustrated in Figure 1.

## 3. Research Method

### 3.1. Survey Method and Pretest

We used a self-report questionnaire to examine the proposed research model empirically. A self-report method refers to an approach in which observation data are provided by participants instead of raters or coders. The method is commonly used in behavioral and management science research. The items of measurement used in the questionnaire were developed based on previous studies. Responses to the various variables related to the perceptions of the individual subjects were measured with 5-point Likert-type scales, ranging from 1 (strongly disagree) to 5 (strongly agree).

Following the public explanation session, the residents were arranged to visit the community center and participated in the survey. Before the interview, the researcher, research assistants, and well-trained interviewers introduced the investigation and invited them to participate in the study. The survey subjects of the questionnaire were those residents who were the end users of a telehealth system from Nantou County, Taiwan. All of these end users had used the telehealth system for at least one month. The participants were interviewed in person. The survey questions were read to participants by interviewers in Chinese and they verbalized their answers or wrote down directly on the survey response scale. The interviews lasted for 20 to 30 min.

**Figure 2:**
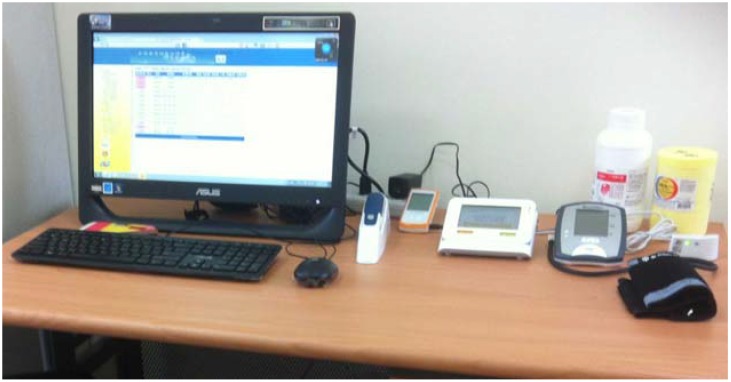
The telehealth system.

The telehealth system was developed and installed by a community hospital in Jhushang township, namely Chu Shang Show Chwan Hospital. The overall telehealth system is shown in Figure 2. It shows that the computer screen with health information system (software) and the peripherals used for physiological recording (hardware). 

The hardware includes weight scale, blood pressure cuff, blood glucose meter, and pulse oximeter. The equipment measures vital signs tailored to residents’ clinical needs, such as weight, blood pressure, blood glucose, heart rate, and oxygen saturation.

Before the study, we conducted a pretest to refine and finalize the survey instruments. A pretest was conducted on 20 selected residents of Nantou County, Taiwan. The respondents were provided with verbal and written feedback on individual items after completing the questionnaire. The scale items were modified according to the feedback of these respondents.

### 3.2. Measurement

The measurement items of the questionnaire in the study were generated from in-depth interviews with senior physicians, nurses, residents who have ever used the telehealth system, and adjusted from the original constructs’ instruments developed by previous studies. The demographic variables include five questions: gender, age, marital status, educational level, and elderly main carer.

Perceived ease of use describes the extent to which a person believes that using the telehealth system will be free of effort. Perceived ease of use was measured with two items which adapted from Davis [52], Davis, Bagozzi, and Warshaw [22], and Venkatesh [49]. A total score ranging from 2 to 10 was obtained by adding the two items. Higher score on the scale were indicative of higher perceived ease of use. The instrument has a high internal consistency (Cronbach’s α = 0.96).

Perceived usefulness is defined as the extent to which a person believes that using the telehealth system will enhance her/his health. Perceived usefulness was measured with three items which adapted from Davis [52], Davis, Bagozzi, and Warshaw [22], and Venkatesh [49]. A total score ranging from 3 to 15 was obtained by adding the three items. Higher score on the scale were indicative of higher perceived usefulness. The instrument has a high internal consistency (Cronbach’s α = 0.99).

Usage intention is the strength of one’s intention to use the telehealth systems. Usage intention was measured with two items which adapted from Davis [52], Davis, Bagozzi, and Warshaw [22], and Venkatesh [49]. A total score ranging from 2 to 10 is obtained by adding the two items. Higher score on the scale are indicative of higher usage intention. The instrument has a high internal consistency (Cronbach’s α = 0.99).

System self-efficacy refers to a belief of one’s capability to use the telehealth systems. System self-efficacy was measured with three items which adapted from Compeau and Higgins [48], Venkatesh [49], and Rahimpour *et al.* [10]. A total score ranging from 3 to 15 was obtained by adding the three items. Higher score on the scale were indicative of higher system self-efficacy. The instrument has a high internal consistency (Cronbach’s α = 0.99).

Social trust refers to the expectation that health-care providers will act competently, fairly, openly, and considerately. Social trust was measured with three items which adapted from Mohseni and Lindstrom [38]. A total score ranging from 3 to 15 was obtained by adding the three items. Higher score on the scale were indicative of higher social trust. The instrument has a high internal consistency (Cronbach’s α = 0.99).

Institutional trust refers to an individual’s trust in the hospital. Institutional trust was measured with three items which adapted from Mohseni and Lindstrom [38]. A total score ranging from 3 to 15 was obtained by adding the three items. Higher score on the scale were indicative of higher institutional trust. The instrument has a high internal consistency (Cronbach’s α = 0.96).

Social participation describes how actively a person has taken part in social free-time activities. Social participation was measured with three items which adapted from Nummela *et al.* [37]. A total score ranging from 3 to 15 was obtained by adding the three items. Higher score on the scale were indicative of higher social participation. The instrument has a high internal consistency (Cronbach’s α = 0.80). 

### 3.3. Statistical Method

For examining the proposed model empirically, structural equation modeling (SEM) was used to validate the model and hypotheses. SEM is a statistical methodology that involves a confirmatory approach to analyzing a structural theory bearing on a particular phenomenon. Typically, this theory represents “causal” processes that generate observations of multiple variables [53]. The data analysis proceeded according to the two-step approach recommended by Anderson and Gerbing [54]. First, the assessment of the measurement model consisting of 10 latent factors included reliability, discriminant validity, and convergent validity of the scales. Second, the structural model was validated individually by considering the series of path relationships linking the 10 constructs.

## 4. Results

### 4.1. Sample Characteristics

Among the 370 subjects recruited, 365 subjects participated in this study. Among the respondents, 217 respondents were women (59.5%) and 148 were men (40.5%). Most of the respondents were aged 71–80 years (41.9%) and held elementary school diplomas (46.8%). Most of the caregivers were married (48.8%). The respondents suffered from at least one chronic disease (71.2%). Table 1 presents descriptive statistics for the seven constructs in this study. The mean scores of seven constructs were all higher than the middle point of the 5-point Likert-type scales and exhibited a reasonable dispersion in their distributions among the ranges. The evidence suggested that the telehealth system was remarkably useful and efficient for the health promotion of the residents, and that they were willing to overcome barriers to using it. 

**Table 1:** Sample Demographics.

Construct	Mean	Standard Deviation	Minimum	Maximum
Social Trust	4.70	0.58	1	5
Institutional Trust	4.48	0.69	2	5
Social participation	4.09	1.05	1	5
System Self-efficacy	3.44	1.47	1	5
Perceived Ease of Use	4.50	0.79	2	5
Perceived Usefulness	4.72	0.52	3	5
Usage Intention	4.59	0.70	2	5

### 4.2. Measurement Model Results

To validate the measurement model, three types of validity were assessed: content validity, convergent validity, and discriminant validity. Content validity was done by interviewing senior system users and pilot-testing the instrument. And the convergent validity was validated by examining Cronbach’s α, composite reliability and average variance extracted from the measures [55]. As shown in Table 2, the Cronbach’s α of every subscale range from 0.80 to 0.99 was above the acceptability value 0.7 [56]. Moreover, the composite reliability values, which ranged from 0.81 to 0.99, and the average variances extracted by our measures, which ranged from 0.59 to 0.99, are all within the commonly accepted range greater than 0.5 [55]. In addition, all measures are significant on their path loadings at the level of 0.001. Therefore, the convergent validities of all constructs are confirmed.

**Table 2:** Construct Reliability and Convergent Validity.

Construct	Cronbach’s α	Composite Reliability	Average Variance Extracted
Social Trust	0.99	0.99	0.98
Institutional Trust	0.96	0.96	0.90
Social participation	0.80	0.81	0.59
System Self-efficacy	0.99	0.99	0.97
Perceived Ease of Use	0.96	0.96	0.92
Perceived Usefulness	0.99	0.99	0.98
Usage Intention	0.99	0.99	0.99

In addition, according to Fornell and Larcker [57], if all variables are standardized, then the squared correlation between two constructs *γ^2^* is equal to the variance shared between these two constructs in the model. The average variance extracted (AVE) of these two constructs, namely *ρ(η)* and *ρ(ε)* can be used to evaluate discriminant validity. To fully satisfy the requirements for discriminant validity, *ρ(η)* > *γ^2^* and *ρ(ε)* > *γ^2^*. That is, the AVE of the construct should be greater than the variance shared between the construct and other constructs in the model. Table 3 lists the squared correlation (*γ^2^*) and AVE (*ρ*) matrix, with the squared correlations among constructs and the AVE on the diagonal. 

**Table 3:** Comparison of Squared Correlation and Average Variance.

	1	2	3	4	5	6	7
1. Social Trust	(0.98)						
2. Institutional Trust	0.41	(0.90)					
3. Social participation	0.29	0.15	(0.59)				
4. System Self-efficacy	0.11	0.08	0.29	(0.97)			
5. Perceived Ease of Use	0.33	0.25	0.26	0.31	(0.92)		
6. Perceived Usefulness	0.35	0.35	0.34	0.15	0.50	(0.98)	
7. Usage Intention	0.33	0.39	0.24	0.16	0.43	0.57	(0.99)

Note: All correlations are significant at the 0.001 level, and diagonal elements are the average variance extracted.

In all cases, the AVE for each construct is larger than the squared correlation of that construct with all other constructs in the model. Therefore, the results confirm that the discriminant validity of constructs in the study is satisfactory.

### 4.3. Structural Model Results

Subsequently, the structural model (which includes hypotheses in addition to the paths between the item and its latent construct) was examined on the measurement model. To validate the structural model, we used AMOS 18.0 to assess the analysis. Several goodness-of-fit statistics were used to access the overall goodness-of-fit of proposed model: the ratio of chi-square to the degree of freedom (*χ*^2^ /d.f.), goodness-of-fit index (GFI), adjusted goodness-of-fit index (AGFI), root mean square error of approximation (RMSEA), root mean square residual (RMR), normed fit index (NFI), relative fit index (RFI), incremental index of fit (IFI), Tucker-Lewis index (TLI), and comparative fit index (CFI). The following indices recommended by Hair * et al.* [49], as the criteria for the model’s evaluation: (1)*χ*^2^ /d.f. should be less than 3, (2) GFI should be more than 0.9, (3) AGFI should be more than 0.9, (4) RMSEA should be less than 0.05, (5) RMR should be less than 0.05, (6) NFI should be more than 0.9, (7) RFI should be more than 0.9, (8) IFI should be more than 0.9, (9) TLI should be more than 0.9, (10) CFI should be more than 0.9. In general, the closer the observed data is to the theoretical model, the better the fit of the model, and the easier it will be to satisfy the thresholds of the above indices. If the threshold of an index cannot be met, it means the model must be modified [58].

As shown in the Table 4, the results of structural equation modeling obtained for the proposed conceptual model revealed that *χ*^2^ /d.f. = 1.284 (*p* < 0.001), GFI = 0.952, AGFI = 0.933, RMSEA = 0.028, RMR = 0.024, NFI = 0.986, RFI = 0.982, IFI = 0.997, TLI = 0.996, and CFI = 0.997. 

These fit statistics of GFI, AGFI, NFI, RFI, IFI, TLI, and CFI were all far greater than the recommended threshold value 0.9. Furthermore, *χ*^2^ /d.f. value was less than the recommended threshold value 5, RMSEA value was less than the recommended threshold value 0.05, and RMR value was less than the recommended threshold value 0.05. Accordingly, the summary of the overall goodness-of-fit indices indicated excellent fit of the model and data. That is, the proposed research model has good fitness.

**Table 4:** Fit Indices for the Structural Model.

Structural Model Statistic	Fit Indices	Recommended Threshold
*χ*^2^	173.287	-
*χ*^2^ / d.f.	1.284	<5
GFI	0.952	>0.9
AGFI	0.933	>0.9
RMSEA	0.028	<0.05
RMR	0.024	<0.05
NFI	0.986	>0.9
RFI	0.982	>0.9
IFI	0.997	>0.9
TLI	0.996	>0.9
CFI	0.997	>0.9

### 4.4. Hypotheses Testing

**Figure 3:**
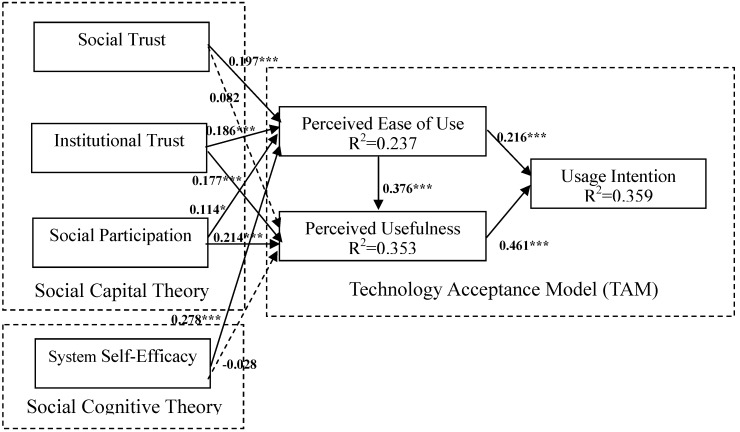
Final Proposed Model.

The results for the structural model with the estimated standardized path coefficients and path significance among the constructs are presented in Figure 3 and Table 5. The estimated standardized path coefficients indicate the strengths of the relationships between the dependent and independent variables. The squared multiple correlations (R^2^) value represents the proportion of variance that is explained based on the predictors of the variables in a model. As predicted, almost all of the proposed hypotheses are supported. As expected, social trust (*β* = 0.197, *p* < 0.001), institutional trust (*β* = 0.186, *p* < 0.001), social participation (*β* = 0.114, *p* < 0.05), and system self-efficacy (*β* = 0.278, *p* < 0.001) significantly affected perceived ease of use, accounting for 23.7% of the variance among the constructs. This means that H1, H3, H5, and H7 are supported. Institutional trust (*β* = 0.177, *p* < 0.001), social participation (*β* = 0.214, *p* < 0.001), and perceived ease of use (*β* = 0.376, *p* < 0.001) significantly affected perceived usefulness, accounting for 35.3% of the variance among the constructs. Thus, H4, H6, and H9 are supported. However, social trust and system self-efficacy affected perceived usefulness nonsignificantly, implying that H2 and H8 were not supported. In addition, perceived ease of use (*β* = 0.216, *p* < 0.001), and perceived usefulness (*β* = 0.461, *p* < 0.001) both significantly affected usage intention, accounting for 35.9% of the variance among the constructs. Thus, H10 and H11 are supported. Table 5 summarizes the results for the hypotheses.

Table 6 lists the standardized indirect and total effects in the proposed model. A direct effect, the influence of one variable on another, is represented in a structural model by a single path. An indirect effect assesses the impact of one variable on another as that variable’s influence works through one or more intervening variables. The total effect of one variable on another is the sum of its direct and indirect effects [59]. As shown in Table 6, perceived usefulness exerted the strongest overall effect on intention to use, followed by perceived ease of use. Perceived ease of use exerted the strongest overall effect on perceived usefulness, followed by social participation. In addition, system self-efficacy had the strongest direct effect on perceived ease of use, followed by social trust.

**Table 5:** Hypotheses Validated Results.

Path	Results	Standardized Path Estimate
H1 Social Trust ➔ Perceived Ease of Use	Supported	0.197 ***
H2 Social Trust ➔ Perceived Usefulness	Not Supported	0.082
H3 Institutional Trust ➔ Perceived Ease of Use	Supported	0.186 ***
H4 Institutional Trust ➔ Perceived Usefulness	Supported	0.177 ***
H5 Social Participation ➔ Perceived Ease of Use	Supported	0.114 *
H6 Social Participation ➔ Perceived Usefulness	Supported	0.214 ***
H7 System Self-Efficacy ➔ Perceived Ease of Use	Supported	0.278 ***
H8 System Self-Efficacy ➔ Perceived Usefulness	Not Supported	-0.028
H9 Perceived Ease of Use ➔ Perceived Usefulness	Supported	0.376 ***
H10 Perceived Ease of Use ➔ Intention to Use	Supported	0.216 ***
H11 Perceived Usefulness ➔ Intention to Use	Supported	0.461 ***

Notes: *** path is significant at the 0.001 level, ** path is significant at the 0.01 level, * path is significant at the 0.05 level.

**Table 6:** Standardized Indirect and Total Effects in the Proposed Model.

Construct	Perceived Ease of Use	Perceived Usefulness	Intention to Use
Social Trust	NA/0.197	0.082/0.156	0.077/0.077
Institutional Trust	NA/0.186	0.177/0.247	0.154/0.154
Social Participation	NA/0.114	0.214/0.257	0.143/0.143
System Self-Efficacy	NA/0.278	−0.028/0.077	0.096/0.096
Perceived Ease of Use	-	NA/0.376	0.173/0.389
Perceived Usefulness	-	-	NA/0.461
Intention to Use	-	-	-

Notes: Number before the slash represent indirect effects, numbers after the slash represent total effects; NA means not applicable.

## 5. Discussion

This study investigated the determinants of behavioral intention toward the telehealth acceptance of elderly rural residents in Taiwan by integrating social capital, system self-efficacy, and the TAM. The results strongly support the proposed behavioral model and provide comprehensive understanding of the relationships among social capital (social trust, institutional trust, and social participation), system self-efficacy, technological factors (perceived ease of use and perceived usefulness), and behavioral intention to use this system. Based on the results of SEM, the hypotheses validate the fit of empirical data and the proposed model. The results show that nearly all of the relationships proposed in the model are supported. In addition, the model demonstrated excellent fit and is thus valuable for evaluating and predicting the behavioral intention toward adopting telehealth. 

However, social trust affected perceived usefulness nonsignificantly. This unexpected finding can be attributed to the interpersonal trusting climate that may not directly affect the perception of usefulness of people. The effect of social trust on perceived usefulness may be mediated by social participation. Nahapiet and Ghoshal [32] argued that the relationships of users can strongly influence their willingness to share information in interactive environments, such as social networks or social participation. For example, through “weak ties” and “friends and friends,” network members can acquire privileged access to relevant health information and knowledge, which improves residents’ perception of usefulness toward telehealth systems. Similarly, system self-efficacy affected perceived usefulness nonsignificantly. Consistent with the results provided in Hasan [8], the effect of system self-efficacy on perceived usefulness was negative (*β* = −0.028). One possible explanation is that people exhibiting high system self-efficacy beliefs may be able to identify the limitations of telehealth systems that may not be immediately obvious to those exhibiting low efficacy beliefs [60].

The results of this study indicate that the elderly rural residents generally reported positive perceptions toward ease of use, usefulness, and intention to use the telehealth system. Moreover, perceived usefulness was the most significantly direct antecedent of behavioral intention. This result is consistent with that presented in Liu, Tsai, and Jang [9], who focused on the acceptance of patients toward a web-based personal health record system. Therefore, the empirical finding of the current study indicates that the elderly rural residents valued the benefits derived from the telehealth system. In addition, Davis, Bagozzi, and Warshaw [22] also found that users would adopt a new IT system if they perceived the system to be useful, even if they disliked it. In other words, how to provide elderly rural residents with independence, security, confidence, quality of life, and the ability to remain in their own homes is considerably crucial in developing telehealth systems [3]. In addition, further investment is necessary to manage the infrastructure to support and sustain the use of such technologies to ensure their safety, security, and reliability [6].

The findings of this study also imply that social capital factors play a key role in perceived ease of use and perceived usefulness. Accordingly, some practices can increase social trust by encouraging interpersonal interactions of residents through, for example, religious communities, sports or dancing clubs, formal or informal meetings, and activities that facilitate the flow of affection within a community. In addition, to improve institutional trust, cultivating equality and a partnership in the doctor-patient relationship is crucial. Health service providers expressing their willingness to refer to and respect patients’ feelings, opinions, and self-knowledge is critical. Social participation encourages the attachment of people in public (*i.e.*, community identity). Kwak, Shah, and Holbert [61] demonstrated that various forms of social associations may strongly contribute to the community participation of people. Developing social associations is essential, because people with stronger psychological ties to their communities are more active in telehealth usage. 

In addition, the findings of this study suggest that system self-efficacy significantly affect ease of use perception. The training courses aimed at improving the system self-efficacy of end users could cause increased user acceptance [16]. In introductory telehealth presentations or training programs, no requirement to possess any knowledge on the telehealth system must be emphasized [10]. Thus, successfully deploying this innovation requires more than merely installing the equipment. The role of specialists and community nurses is vital, because the telehealth system is used to enhance health services for rural residents [3]. Local service professionals and remote call centers should provide real-time support cooperatively to users when they encounter any obstacles during operating processes. These proactive design interventions may maximize the acceptance of rural residents to use the technology and promote its effective diffusion [10]. 

In this study, behavioral intention was used as dependent variable instead of actual usage behavior. Future studies that incorporate actual telehealth usage into the research model would enable a more comprehensive examination of the integrative model in explaining or predicting telehealth acceptance by residents.

## 6. Conclusions

This study integrated two sociopsychological theories, social capital theory and social cognitive theory, with a widely used IS technology acceptance model (*i.e.*, the TAM) to provide a comprehensive behavioral model for understanding elderly rural residents’ intention toward using telehealth systems. The framework was extended from the original TAM by considering the relationships among social capital factors (social trust, institutional trust, and social participation), technological factors (perceived ease of use and perceived usefulness), social cognitive factors (system self-efficacy), and behavioral intention to use the system. The proposed model has been proven to be valuable for evaluating and predicting the behavioral intention of telehealth system because it provides an integrative perspective that prompts researchers and practitioners to pay attention to the interdependence of these aspects. The integrative psychosocial-technological viewpoint implies that hospital managers should consider these key factors simultaneously.

Recently, small sample sizes (typically less than 100) have limited the generalizability of most previous telemedicine/telecare/telehealth studies, causing barriers to knowledge development in the telemedicine/telecare/telehealth area [62]. The large sample size of the current empirical study (365 respondents) can overcome methodological concerns of generalizability and provide valuable insights into the innovative technology development.

The delivery of health care is being transformed by advances in e-health and by an empowered, computer-literate public. However, interactive IS developed for both patients and physicians remain at the early stages of development [9]. This study could be a useful foundation for exploring the implementation of telehealth systems in Taiwan. Future research could continue to explore other factors that influence decisions for adopting telehealth systems. We believe that the proposed integrated model will provide valuable and informative contributions to practitioners and scholars. 
